# Socioeconomic disparities and nutritional aging: how food insecurity and financial hardship accelerate health decline in older adults

**DOI:** 10.3389/fnut.2026.1833736

**Published:** 2026-06-08

**Authors:** Claudia Reytor-González, Martín Campuzano-Donoso, Náthaly-Mercedes Román-Galeano, Janeth Castano Jimenez, Carolina Paz-Yépez, Martha Montalvan, Daniel Simancas-Racines

**Affiliations:** 1 Facultad de Ciencias de la Salud y Bienestar Humano, Universidad Tecnológica Indoamérica, Ambato, Ecuador; 2 Facultad de Ciencias Médicas, de la Salud y la Vida, Universidad Internacional del Ecuador UIDE, Quito, Ecuador; 3 Emergency Medicine Department, Indiana University School of Medicine, Indianapolis, IN, United States; 4 Instituto de Investigación, Universidad Agraria del Ecuador, Guayaquil, Ecuador; 5 Escuela de Medicina, Universidad Espíritu Santo, Samborondón, Ecuador

**Keywords:** food insecurity, healthcare, malnutrition, nutritional aging, nutritional consequences, older adults, socioeconomic disparities

## Abstract

This narrative review examines how socioeconomic disparities accelerate health decline in older adults. As the global population ages, older age represents a major risk factor for chronic diseases, frailty, and disability, particularly in socially disadvantaged groups. While adequate nutrition is essential to healthy aging, approximately 28% of the global population experiences moderate or severe food insecurity, with older adults disproportionately affected in resource-limited settings. Food insecurity and financial hardship are consistently associated with poorer diet quality, including lower Healthy Eating Index scores (e.g., ~51 vs. >57 in food-secure peers), and increased risk of adverse outcomes such as sarcopenia, frailty, and cognitive decline. Notably, food-insecure older adults are also more likely to experience overweight or obesity (OR ≈ 1.29), reflecting reliance on energy-dense, nutrient-poor diets. These disparities are shaped by structural and socioeconomic factors, including low income, limited education, inadequate social safety nets, and inequitable neighborhood food environments. Addressing these challenges requires a shift from individual-level interventions to systemic, interdisciplinary, and culturally-tailored approaches.

## 1 Introduction

Worldwide, demographic shifts are producing an unprecedented increase in older adults. As of 2020, the global population aged 60 years and over surpassed the number of children under five, and is projected to double by 2050, reaching approximately 2.1 billion globally (1). This demographic trend carries profound implications for global health systems: ageing is the primary risk factor for chronic, non-communicable diseases, multimorbidity, frailty, and disability (2). Rising longevity does not uniformly equate to extended health span; instead, increases in age-related diseases such as cardiovascular disease, diabetes, cancer, malnutrition and musculoskeletal and immune disorders are intensifying demands on health infrastructure and long-term care systems (3–5).

Particularly in low- and middle-income countries, where population ageing is happening rapidly, health systems often lack the financial and organizational capacity to respond (1). Even in high-income countries, age-related illnesses and functional decline are expected to sharply escalate public health costs, impair productivity, and burden economies (6). Thus, achieving healthy aging has become a global priority, recognized by the UN and WHO frameworks around intrinsic capacity, encompassing physical, cognitive, sensory, vitality, and psychological domains, as the desired outcome of aging (7, 8).

Nutrition plays a central role in preventing age-related functional decline (6). Both undernutrition (including micronutrient deficiencies, insufficient protein, and energy intake) and overnutrition (overweight, obesity, diet-related disease) contribute to sarcopenia, frailty, cognitive impairment, poorer quality of life, and increased mortality among older adults (3, 9, 10). Worldwide estimates indicate that roughly one in four adults aged 65 years or older are malnourished or at risk of malnutrition (11).

Longitudinal and global studies consistently link poor dietary patterns with accelerated physical and mental decline. For example, diets high in plant-based foods, whole grains, legumes, unsaturated fats, and low in processed meats and sugary beverages are associated with higher odds of successful aging (12). Evidence from systematic reviews and meta-analyses further supports the protective role of high-quality dietary patterns in reducing frailty, cognitive decline, and mortality risk (13). Intervention strategies emphasize early detection via routine nutritional screening (e.g., MUST, SARC-F, R-MAPP tools), individualized dietary counselling, food fortification, supplementation, and ensuring sufficient protein and micronutrient intake to maintain muscle mass and function (14, 15). Among these determinants, food insecurity emerges as a central mechanism linking socioeconomic disadvantage with nutritional decline in older adults.

Food insecurity is defined as limited or uncertain access to sufficient, safe, and nutritious food due to financial constraints (16, 17). Food insecurity frequently includes disruptions to normal eating patterns, reliance on low-quality diets driven by affordability constraints, skipped meals, and reduced dietary diversity, thus acting as a key driver of malnutrition and impaired health (18). It is also associated with increased vulnerability to micronutrient deficiencies and diet-related chronic diseases, particularly among already disadvantaged populations (19). Over time, persistent food insecurity can exacerbate health inequalities by limiting access to nutritionally adequate diets and undermining overall physical and mental well-being. Globally, in 2023 approximately 2.33 billion people experienced moderate or severe food insecurity, representing 28.9% of the world’s population, with over 864 million individuals enduring severe food deprivation (20–23). Among older adults, prevalence varies by context. In the U.S., food insecurity among adults aged 60 + rose from around 5.5% in 2007 to over 12% by 2016, and subsequently stabilized around 7-9% in recent years, with higher rates among older adults living alone, those with fixed incomes, chronic illness, or low education (1, 16). Globally representative data from six LMICs show moderate to severe food insecurity affecting approximately 6.7 and 5.0%, respectively, among adults ≥ 65 years, with the poorest older cohorts facing approximately twofold higher odds of sarcopenia and worse outcomes (24).

Economic constraints and income insufficiency overlap strongly with food security. In diverse settings, from Thailand to Ethiopia to the U.S., older adults reporting income insufficiency, persistent debt, dissatisfaction with finances, or material deprivation are significantly more likely to be food insecure (1). National estimates in Thailand found roughly 29% of older adults lacked food security, with financial stressors (sometimes/often income problems, debt) strongly predictive of insecure food status (1). In the U.S., older adults with fixed incomes and mounting healthcare or housing costs are especially vulnerable, with under-enrollment in governmental assistance programs such as the Supplemental Nutrition Assistance Program (SNAP) exacerbating gaps (25). Overall, this narrative review examines how socioeconomic disparities, including food insecurity and financial hardship, undermine nutritional status and accelerate health decline in older adults.

## 2 Methodology

This study was conducted as a narrative review to synthesize the current evidence on the relationship between socioeconomic disparities, food insecurity, and nutritional aging in older adults. A structured literature search was performed using databases including PubMed, Scopus, and Web of Science, focusing on studies published in English that addressed food insecurity, economic hardship, and age-related health outcomes. Studies were selected based on their relevance to the conceptual framework of the review, prioritizing epidemiological, clinical, and public health research that provided insight into the mechanisms linking socioeconomic factors and nutritional status in older populations. Given the complexity and multidimensional nature of the topic, a narrative approach was chosen to integrate evidence from epidemiological, clinical, and public health perspectives. This approach enables the identification of key themes, pathways, and gaps in the literature, while acknowledging that the review is not exhaustive and does not follow a formal systematic review protocol.

## 3 Socioeconomic determinants of nutrition in older adults

The socioeconomic determinants of nutrition in older adults operate across multiple levels: structural, environmental, and individual. These determinants influence food security through key dimensions such as food availability, economic and physical access, utilization, and stability. Importantly, these determinants do not act in isolation but interact to influence dietary behaviors and health outcomes in later life.

### 3.1 Income, education, and employment status in aging populations

Income levels exert a major influence on older adults’ nutritional health. A growing body of evidence indicates that lower income is strongly associated with reduced diet quality, increased prevalence of food insecurity, higher risk of malnutrition, and higher rates of chronic conditions (26). For instance, a systematic review found strong associations between low income, particularly in rural environments, and the risk of malnutrition among older adults (27). Among lower socioeconomic strata, economically disadvantaged older adults experience diminished ability to purchase nutrient-dense foods, thereby increasing the risk of sarcopenia, frailty, and functional disability (28). In line, meta-analyses have further demonstrated that socioeconomic disadvantage is associated with increased risk of frailty and sarcopenia, partly mediated by inadequate protein intake, poor diet quality, and reduced access to healthcare resources (29). Moreover, educational attainment, as a component of socioeconomic position, also shapes dietary behaviors. Lower education often coincides with poorer health literacy, weaker understanding of nutritional recommendations, and reduced ability in preparing balanced meals (30). Among older adults, low education remains a significant predictor of malnutrition, independently of income and living arrangement (27).

Regarding employment status in later life, a systematic review (31) examined health outcomes associated with extended working life in older adults. The review found that continued employment beyond retirement age, particularly in part-time or flexible roles with high job quality, was generally associated with neutral to beneficial physical health outcomes, especially for men. Such employment can help sustain income, reduce social isolation, and maintain physical function, thereby supporting economics and physical access to food, meal preparation, and dietary adequacy in older age. Conversely, working full-time in physically demanding or low-quality jobs was linked to worse health outcomes, potentially exacerbating stress and physical decline, which can impair appetite, limit the ability to obtain and prepare healthy foods, and increase the risk of malnutrition. In addition, gender differences were also noted: while men benefitted more consistently from late-life employment, women experienced more variable outcomes, with those balancing caregiving and part-time work showing lower frailty later in life. These findings highlight the complex and multidimensional role of socioeconomic factors across the life course, where income, education, and employment interact to shape nutritional risk and health trajectories in older age.

### 3.2 The role of social safety nets and retirement income

Social safety nets, including pensions, social security, and cash transfers, play a protective role against food insecurity and undernutrition in older adults by stabilizing income and reducing economic barriers to food access. Evidence from systematic reviews indicates that social protection programs are associated with improved food security and dietary outcomes among vulnerable populations in developing counties (32). On the other hand, evidence from high-income settings, such as Canada, shows that while government transfers reduce the severity of food insecurity, they do not fully eliminate it, indicating persistent gaps in nutritional adequacy (33). These findings suggest that while social support mitigates extreme food deprivation, it may be insufficient for ensuring nutritional adequacy. In line, Khan et al. conducted a household survey in Torghar, Northern Khyber Pakhtunkhwa, Pakistan, assessing how various social safety net mechanisms affect household food security (34). Their bivariate and multivariate analyses revealed that when effectively administered, such transfers significantly improved food access and nutritional sufficiency. However, systemic impediments, such as bureaucratic complexity, political favoritism, nepotism, and corruption, severely undermined the reach and effectiveness of the safety nets. Although the study focused on younger-age cohorts, the implications are clear: well-targeted social assistance delivery, particularly when not compromised by corruption and logistical faults, can directly increase food availability and diet quality among vulnerable households, including older adults who often rely on fixed or insufficient retirement income. By reducing economic constraints on food purchasing, dependable safety-net income supports the ability to acquire nutrient-dense foods, buffer against seasonal shortages, and avoid reliance on low-cost energy-dense diets. Evidence further suggests that cash transfer programs can improve dietary diversity and caloric adequacy, although their impact on nutritional quality and health outcomes may vary depending on program design and implementation (35, 36). However, where governance failures persist, these programs fail to protect nutritional status, especially in households lacking alternative sources of income or savings, the very conditions rural older adults often face (37).

### 3.3 Neighborhood food environments and access to healthy foods

Beyond individual socioeconomic position, neighborhood context critically shapes dietary outcomes in older adults. Limited access to affordable, nutritious foods—often described as “food deserts,” is associated with both poorer diet quality and adverse health outcomes. For example, a recent longitudinal study of nearly 5,000 U.S. urban seniors found that living in neighborhoods with both low economic and physical access to food and low income was associated with significantly accelerated cognitive decline (*β* = − 0.19; 95% CI = − 0.32, − 0.05 per year) (38).

Neighborhood food environments also influence obesity risk, as noted in a recent systematic review and meta-analysis (39–41). A meta-analysis on this topic, which synthesized evidence from 103 studies using spatial and statistical approaches, found that, proximity to fast-food outlets is significantly associated with higher obesity rates, while access to supermarkets and fresh produce outlets is linked to lower obesity rates (39). However, no consistent associations were observed for other food outlet types, such as restaurants or convenience stores, highlighting heterogeneity in environmental exposures. These findings underscore the limitations of relying solely on zoning policies and suggest that more comprehensive, multi-level interventions are needed to promote equitable access to healthy foods. Overall, these insights provide important implications for policymakers and public health strategies aimed at reducing obesity and diet-related non-communicable diseases (42, 43).

Social capital and networks also play a role. A review of social capital’s influence on food security shows that stronger community ties are associated with increased food sharing, dietary knowledge exchange, and access to nutritious food (44). Among older adults, shared meals and social engagement can enhance diet diversity and nutritional outcomes, highlighting the interplay between the local infrastructure and the social environment (45). Together, these findings underscore that neighborhood context influences nutrition through both physical infrastructure and social dynamics.

### 3.4 Impact of inflation and cost of living on food affordability

In the current global economic context, escalating food prices and increasing living costs are significantly constraining access to nutritionally adequate diets, particularly among older adults who rely on fixed or limited incomes. These economic pressures disproportionately affect vulnerable populations, whose ability to adapt to price fluctuations to price fluctuations is often limited. Recent empirical research indicates that individuals experiencing food insecurity are especially sensitive to food price inflation, facing greater challenges in maintaining stable access to sufficient and nutritious food compared to food-secure populations (46). Evidence from recent systematic reviews further suggests that food price volatility disproportionately affects low-income households, amplifying existing inequalities in diet quality and food access (47). Consequently, rising food inflation not only exacerbates existing inequalities in food access but also intensifies the risk of poor dietary quality and adverse health outcomes among economically vulnerable groups. Headey et al. demonstrate that sharp food price inflation, especially in low- and middle-income countries during crises like the COVID-19 pandemic and the war in Ukraine, led to a rapid decline of 20–30% in real wages, when measured as “food wages” (unskilled daily wages deflated by a food-price index) (48). These disruptions have undermined the stability and resilience of global food markets. The conflict has also intensified economic pressures, contributing to rising food prices and reduced purchasing power, which undermines households’ economic access to food (49). Notably, declines in food security occurred within a short time frame, significantly eroding purchasing power among both urban and rural poor populations, who allocate a large proportion of their income to food. As a result, households often shift toward inexpensive, energy-dense, nutrient-poor foods, leading to reduced dietary quality and increased risk of chronic disease (50–53).

Several socioeconomic and structural determinants shape food affordability and nutritional outcomes, particularly for older adults. Table 1 summarizes key factors such as income, education, employment, safety nets, and neighborhood access, highlighting their effects on diet quality and health. In this context, older adults who often experience persistent financial limitations and lack of buffer savings, this trade-off becomes particularly harmful. Rising housing, transport, and medical costs further restrict food budgets, leading to in meals skipping or reliance on economically constrained dietary patterns (26). Table 1 summarizes key factors such as income, education, employment, safety nets, and neighborhood access, highlighting their effects on diet quality and health.

**Table 1:** Socioeconomic determinants of food access and nutritional outcomes in older adults (123, 148–150).

Determinant	Evidence	Effect on Nutrition
Income	Lower income is consistently associated with reduced consumption of healthy foods and increased reliance on processed foods	Increases risk of malnutrition, obesity, chronic disease risk
Education	High education attainment is associated with improved diet quality by ~30%	Improves health literacy, dietary choice, overall diet quality
Employment and pension status	Loss of employment/pension reduces food budget and poorer diet quality	Less financial resilience, increases risk of food insecurity
Social safety nets	Pensions and OAA meals reduce, but do not eliminate, food insecurity (18–21%)	Increase nutrient intake, yet coverage and adequacy limited
Neighborhood food environment	Low-access, low-income areas accelerate cognitive decline (β = –0.19/year)	Limits healthy food availability, increases risk of diet-related diseases
Inflation and cost-of-living	10.8% food inflation; many skip meals; COLA insufficient	Reduces food quantity/quality; increases healthcare trade-offs with essential expenses

## 4 Food insecurity in late life

Building on the socioeconomic determinants outlined above, food insecurity in older adults reflects the combined influence of economic, physical, and social constraints on food access and utilization. Food insecurity is defined by the United Nations as a situation in which individuals or households lack consistent access to sufficient, safe, and nutritionally adequate food that meets dietary needs and personal preferences for an active and healthy life (54). Food insecurity is a multidimensional construct encompassing economic access, physical access, food utilization, and stability over time. In older populations, standard tools such as the USDA Household Food Security Survey Module (HFSSM) primarily capture economic dimensions (e.g., difficulty affording adequate food), but they may fail to detect age-specific limitations (55–57). This dual dimension framework highlights that food insecurity in older adults cannot be fully understood through economic measures alone. However, the measurement of food insecurity in older adults remains heterogeneous across studies, with tools such as the USDA HFSSM primarily capturing economic dimensions while often overlooking functional and age-specific barriers. This variability limits comparability across studies and may lead to underestimation of the true burden of food insecurity in aging populations.

Recent advances incorporate a dual-dimension approach: combining economic constraints with food-related physical functioning limitations (e.g., difficulty shopping, preparing food, chewing) to better capture food insecurity in later life. For instance, NHANES-based research (2013–2018) showed that approximately 25% of adults ≥ 60 years had physical barriers to food access even if they were economically secure; those who experienced both economic insecurity and physical limitations had the lowest Healthy Eating Index (HEI-2015 scores of 51.7) and highest depression scores (6.9) compared to fully secure peers (57). Cross-classification frameworks now differentiate four groups: food secure; economic food insecure; physically constrained food insecure; and combined (dual-dimension) food insecurity (54). Notably, much of this evidence is derived from cross-sectional analyses, which limits the ability to establish temporal or causal relationships between food insecurity and health outcomes. Furthermore, differences in how physical limitations are defined and measured across datasets introduce additional variability in estimates.

Prevalence trends vary by region, survey tool, and definition. Globally, approximately 28% of the population experienced moderate or severe food insecurity in 2024, equivalent to 2.3 billion individuals according to the United Nations (58). While this is a global average spanning all ages, older adults are often disproportionately affected in low resource settings. These prevalence estimates should be interpreted with caution, as differences in survey methodologies, definitions of food insecurity, and population characteristics across regions contribute to substantial heterogeneity in reported rates. At a national level in the United States, data from NHANES (2007–2012) indicate that approximately 8% of adults aged 60 + experienced marginal food security, with an additional 5% reporting food insecurity (25). Disparities are notable: younger older adults (60-64 years) exhibit higher rates; non-Hispanic Black and Hispanic older adults face significantly elevated risks compared with non-Hispanic Whites; lower income, less education, and non-partnered status also correlate with higher prevalence (25). In a European cohort (Portugal) of adults ≥ 65 years, about 23% reported food insecurity, with 16.3% low, 4.8% moderate, and 2.0% severe food insecurity; women and those aged 70–74 were most affected (59). In Latin America, frailty and household food hardship among older adults align strongly: for example, in Mexican adults ≥ 60 years, moderate/severe insecurity was associated with significantly increased odds of frailty (OR 2·41 (95 % CI 2·03, 2·86, *p* < 0·001)) (60). Global projections indicate that malnutrition among older adults increased from 2.5% of total cases in 1990 to 5.3% in 2021, and although absolute numbers remain lower than for children, this trend reflects stagnation in poverty reduction among elderly populations and continued food insecurity following the post-Great Recession (61). Overall, while these studies consistently demonstrate an association between food insecurity and adverse health outcomes in older adults, variability in study design, population characteristics, and measurement tools complicates direct comparisons and may influence the strength of observed relationships.

### 4.1 Causes of food insecurity in older age

Fixed or limited income is a major determinant of food insecurity in late life. Older individuals living primarily on pensions or Social Security often have insufficient funds to maintain a nutritious diet (62). Out-of-pocket healthcare expenses (medications, treatments) frequently compete with food budgets, leading some seniors reduce food expenditures to pay medical bills. Financial instability and lack of liquid reserves further exacerbate vulnerability to food hardship (63).

In addition to financial constraints, physical frailty, disabilities, dental problems, visual or mobility impairments impair older individuals’ ability to shop, prepare, or consume food (54). Evidence from NHANES indicates that older adults with four or more physical limitations are almost three times more likely to report very low food security (54, 64). These limitations reduce independence in accessing and utilizing food, compounding economic hardship.

Beyond physical limitations, social isolation, including living alone or lacking familial support, increases the risk of food insecurity. Those widowed, separated, or divorced are more likely to encounter both economic and physical barriers to sustaining adequate nutrition (65). Isolation reduces opportunities to share meals, and to pool resources and weakens community support that can buffer food hardship.

In parallel, chronic illnesses amplify food insecurity risk: functional limitations from arthritis, diabetes, arthritis or cognitive impairment hinder food access; management needs intensify financial burden; and depression or anxiety, as shown in NHANES, are significantly more common in marginal or food-insecure seniors (25). Notably, dual exposure to economic and physical food insecurity is associated with highest depression scores (65, 66). Although these factors are consistently associated with food insecurity, most evidence remains observational and may be subject to residual confounding, particularly from overlapping socioeconomic and health-related variables.

### 4.2 Coping strategies and their nutritional consequences

Older adults employ various coping mechanisms when facing food insecurity; however, many of these strategies are associated with adverse nutritional and health outcomes. Analyses from Mexican older adults reveal that respondents frequently resorted to skipping breakfast, lunch, or dinner, eating less than needed, or even not eating when hungry (67). For example, among severely food insecure individuals, 36.8% report eating less than they should, 25.6% skip meals, and 25.9% were hungry but did not eat (60). As a consequence, these strategies are associated with increased risk of undernutrition, weight loss, sarcopenia, and frailty. Evidence from systematic reviews further indicates that food-insecure individuals are significantly more likely to engage in meal skipping and portion reduction, behaviors consistently associated with increased risk of malnutrition and functional decline in older populations (68). In addition to meal reduction, financially-strained older adults often select inexpensive, calorie-dense, nutrient-poor foods (e.g., refined carbohydrates, processed snacks) to stretch limited budgets. This compensatory behavior fosters paradoxical coexistence of obesity with micronutrient deficiencies and poor diet quality. Moreover, in individuals with chronic illness, such diets undermine disease management and amplify morbidity (42, 69, 70). Meta-analytic evidence suggests that economic constraints are associated with lower intake of protein, fruits, and vegetables, alongside higher consumption of ultra-processed foods, thereby contributing to both sarcopenic and metabolic risk profiles (71).

Consistent with these patterns, food insecure older adults tend to exhibit lower dietary diversity: fewer servings of fruits, vegetables, lean proteins, and whole grains. NHANES-linked studies show that combined economic and functional food insecurity yields lowest HEI-2015 score (51.7 vs. 57 + in secure peers) (57). In turn, reduced diet quality correlates with nutritional deficiencies, cognitive decline, and reduced physical functioning.

**Figure 1:**
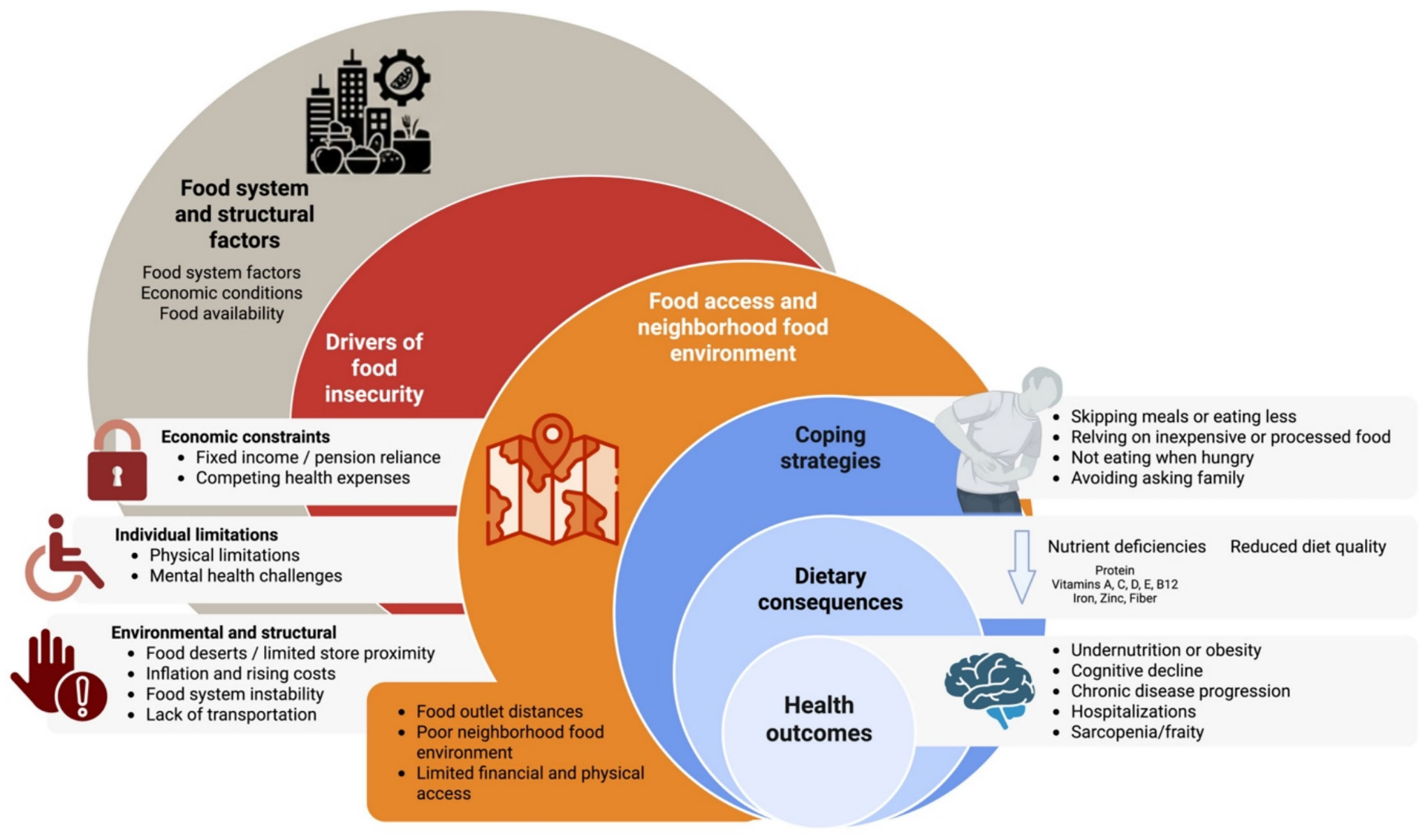
Pathways from food insecurity to health consequences in older adults (123, 146, 147).

At the same time, some older adults cope by relying on family, friends, or informal networks for food support, or participating in community meal programs (e.g., meal delivery) (72). While such interventions can partially alleviate food hardship, uptake is limited and may not be nutritionally sufficient. Furthermore, seniors often under-report needs or avoid assistance due to stigma, limited mobility, or program access barriers (73–75). It is important to note that evidence on coping strategies is often derived from self-reported data, which may be influenced by recall bias and social desirability, potentially underestimating the extent and severity of food insecurity-related behaviors. Figure 1 summarizes the multi-level pathways through which economic, physical, and social constraints shape coping behaviors, dietary patterns, and the resultant health risks in food-insecure older adults.

## 5 Nutritional consequences of food insecurity and financial strain

Food insecurity and financial hardship in older adults are consistently associated with poor diet quality and nutrient shortfalls. Numerous studies show that food-insecure seniors consume substantially less high-quality protein, dietary fiber, and key micronutrients, such as vitamins B-complex, A, C, and E, as well as iron, zinc, and omega-3 fatty acids, than their food-secure peers (76–79). Such micronutrient inadequacies have been consistently documented as key mediators linking food insecurity to adverse health outcomes in aging populations as deficiencies, particularly in antioxidants such as vitamins C and E and B vitamins, not only compromise immune and metabolic health but also progressively undermine cognitive function over time (80). However, these findings are largely based on observational studies, and dietary intake assessments are often subject to measurement error and recall bias, which may affect the precision of estimated nutrient deficiencies.

Regarding the macronutrient intake, protein are often below recommended thresholds: a lot of evidence indicates that inadequate protein intake is strongly associated with increased risk of sarcopenia and physical decline in older adults. While 0.36 g/lb. (0.8 g/kg) is a baseline, older adults require as much as 0.45–0.54 g/lb. (1.0–1.2 g/kg) per day to overcome anabolic resistance and preserve muscle mass (81). Yet surveys indicate up to 30% of seniors in high-income countries fail to meet even the minimum requirement. Similarly, fiber intake is often diminished, as consumption of fruits, vegetables, and whole grains declines under financial constraints. Data from the U.S. show grocery inflation disproportionately affected fresh fruits and vegetables, leading to cuts in these categories in lower-income households (50). Likewise, deficits in zinc, iron, and B12 are common among older food-insecure adults, contributing to anemia, immune dysfunction, and frailty risk (82).

As a result, these dietary inadequacies contribute to increased rates of undernutrition, sarcopenia, and frailty. Longitudinal evidence from older Mexican adult’s links moderate or severe food insecurity to significantly increased incidence of sarcopenia, including severe forms, over a 4–12-year follow-up period (83). Severe food insecurity almost doubled the odds for sarcopenia and severe sarcopenia (OR = 1.91; 95% CI: 1.24–2.94) (83). Consistent with these findings, systematic reviews demonstrate that higher habitual protein intake, especially plant protein, is protective against frailty across diverse cohorts (84). In multiple cohorts (Finland, SHARE, Newcastle 85+), higher habitual protein consumption was associated with lower incident frailty (85). Moreover, frailty prevalence increases incrementally with food insecurity severity: older adults with moderate food insecurity had 2.02 odds of frailty and with severe insecurity 2.41 odds (Mexican cohort) (60). Low dietary diversity, insufficient protein, and micronutrient deficiencies may impair muscle protein synthesis, contributing to loss of lean mass, decreased strength, and increased risk of falls and disability (86–88). While longitudinal studies provide stronger evidence of association, causality remains difficult to establish due to potential confounding factors such as baseline health status, physical activity, and comorbidities.

In resource-constrained settings, food insecurity is related to a double burden: undernutrition in lean seniors and obesity in others, both of which are risk factors for sarcopenia and functional decline (89). The fact that food insecurity is associated with overweight and obesity among older adults, is a phenomenon often termed the “obesity paradox” in low-SEP populations (89–91). A systematic review and meta-analysis up to February 2024 found that food-insecure seniors were 1.29 times more likely to be overweight or obese than food-secure peers (95% CI 1.28-1.30, *p* < 0.001) (92). This paradox is commonly attributed to reliance on inexpensive, energy-dense, nutrient—poor foods, which provide sufficient caloric intake but fail to meet nutritional requirements. In line with this, evidence from systematic reviews [e.g., Carvajal-Aldaz et al. (93)] confirms a consistent association between food insecurity and obesity, particularly among women in high-income settings. The paradox arises because limited budgets drive reliance on inexpensive, calorie-dense foods that provide satiation but lack essential nutrients. These dietary patterns result in adiposity alongside micronutrient deficiencies, contributing to conditions like sarcopenic obesity, where excess weight coexists with low muscle mass (94, 95). Though causal pathways remain under investigation, Carvajal-Aldaz et al. underscore that food insecurity does not simply reflect underconsumption but often entails distorted dietary choices shaped by affordability constraints, with profound implications for metabolic health and functional decline. Additionally, the relationship between food insecurity and obesity appears to vary by sex, socioeconomic context, and geographic region, suggesting that underlying mechanisms may differ across populations.

Beyond physical health, food insecurity in older adulthood is associated with accelerated cognitive decline, particularly in executive function. NHANES-based analyses further corroborate that food insecurity is associated with lower scores in processing speed, sustained attention, verbal fluency, and working memory, relationships that remain significant after adjusting for income and education (76, 96). Mechanistic pathways include inadequate intake of omega-3 fatty acids, antioxidants, B vitamins, zinc, and iron, as well as increased oxidative and psychosocial stress impairing hippocampal function (80). In low- and middle-income countries, older adults experiencing moderate or severe food insecurity had 2.5–3.9 times higher odds of mild cognitive impairment (MCI), particularly pronounced among those aged ≥65 years (97). Nonetheless, inconsistencies in cognitive assessment tools and differences in adjustment for socioeconomic and health-related confounders across studies may contribute to variability in reported associations.

Furthermore, food insecurity is also linked with increased hospitalizations in older adults. Analysis of Health and Retirement Study data demonstrated a bidirectional relationship: food-insecure seniors were more likely to be hospitalized, and past hospitalizations increased subsequent food insecurity risk (98). These bidirectional dynamic highlights a reinforcing cycle between poor health and economic vulnerability. Several mechanisms explain this relationship, including poor dietary management of chronic disease, depression, poor medication adherence, and functional decline. Nutritional compromise exacerbates the progression of chronic conditions: diets high in refined carbohydrates and saturated fats and low in high-quality protein and micronutrients impair glycemic control, lipid profiles, and blood pressure regulation, thereby worsening diabetes, hypertension, cardiovascular disease, and dyslipidemia (89, 99). Taken together, the coexistence of poor diet quality, metabolic dysregulation, and sarcopenia contributes to the development of sarcopenic obesity, a phenotype characterized by excess fat mass and diminished muscle strength. This condition accelerates functional decline, increases fall risk, worsens frailty trajectories, and raises the likelihood of institutionalization (89). Over time, cumulative nutritional deficits further exacerbate disability and increase mortality risk, underscoring the long-term clinical impact of food insecurity in aging populations.

Overall, although the evidence consistently links food insecurity with adverse nutritional and health outcomes in older adults, the literature is characterized by considerable heterogeneity in study design, measurement approaches, and population characteristics. Most studies are observational, limiting causal inference, and residual confounding cannot be excluded. These limitations highlight the need for more longitudinal and intervention-based research to better elucidate causal pathways and inform targeted public health strategies.

## 6 Intersectionality: race, gender and marginalized groups

Nutritional decline among older adults is significantly influenced by the intersection of race, ethnicity, immigration status, and gender. These overlapping identities create distinct vulnerabilities that contribute to unequal health outcomes (100). For instance, a cohort study demonstrated that older adults residing in low-income, urban areas with poor access to nutritious food experienced a more rapid decline in cognitive function, highlighting the critical role that neighborhood food environments play in shaping health disparities (38). However, much of the evidence in this area remains observational and context-specific, limiting generalizability across different populations and settings.

A growing body of evidence suggests that structural racism is a fundamental driver of these disparities, particularly among ethnic minority groups. Historical policies such as redlining have led to decades of disinvestment in predominantly Black and Latinx neighborhoods, restricting access to affordable, healthy foods and increasing reliance on fast food and ultra-processed options (101, 102). Such inequitable food environments contribute to poor diet quality and increased prevalence of obesity, hypertension, and diabetes, which are established risk factors for cognitive decline and dementia (38).

In this context, accelerated aging is emerging as a key, modifiable contributor to health disparities. Racialized experiences, especially among Black adults, have been linked to faster biological aging, often reflected in a greater gap between biological and chronological age (103). This process is thought to be driven by chronic exposure to systemic and psychosocial stressors (e.g., economic instability, limited educational opportunities, and adverse living environments), which cumulatively increase the risk of mortality and age-related diseases (104). Mitochondrial dysfunction, a key hallmark of accelerated aging, is closely linked to a wide range of chronic diseases including cardiovascular disease, cancer, neurodegenerative disorders, diabetes, obesity, and systemic inflammation (105).

Addressing these disparities requires systemic, rather than solely individual-level, interventions. Policy reforms must address the root causes of structural inequities to promote equitable food access (106). For instance, a qualitative study across four Mexican states found that food insecurity, primarily driven by unstable income, affected households at all marginalization levels. Concerns about food scarcity peaked near the end of pay cycles, prompting varied coping mechanisms. These findings underscore the importance of dynamic, context-sensitive assessments of food insecurity that reflect lived experiences rather than static definitions (107). Importantly, food insecurity extends beyond physical access to food and reflects the structural absence of affordable, nutritious, and culturally appropriate options.

The concept of intersectional burdens further underscores how overlapping social identities, such as race, class, gender, age, and disability, compound barriers to accessing public resources. These intersecting disadvantages are frequently both racialized and gendered, disproportionately limiting access to support among women and minority populations (108, 109). For example, 2019 data from the SNAP show that 92% of single-adult households receiving benefits were headed by women, pointing to broader structural inequalities in caregiving responsibilities, employment opportunities, and access to healthcare (110). Accordingly, effective interventions must account for these layered barriers; for instance, simplifying SNAP enrollment processes should also address cultural and gender-specific constraints affecting access and utilization.

In addition, older immigrants often strive to preserve traditional dietary practices, recognizing the deep cultural and emotional significance of food. A systematic review found that maintaining familiar eating habits serves as a source of comfort and identity for aging immigrants (111). However, limited access to culturally appropriate foods can lead to partial meal consumption or food rejection, negatively impacting nutritional intake and overall health (111). As caregiving increasingly shifts from family-based to institutional systems, culturally sensitive nutrition services become essential to meet the specific needs of older immigrant populations (112).

From a broader perspective, economic stability, a core social determinant of health (SDOH), is fundamental to securing essentials such as housing, healthcare, and nutritious food (113). Yet, nearly 10% of older Americans live in poverty (114). Poverty imposes both physical and psychological stressors that erode well-being and exacerbate health disparities (106, 115). Older adults relying on fixed incomes, for instance Social Security, are particularly vulnerable to rising living costs and potential reductions in future benefits (116). Nutrition disparities are deeply rooted in broader SDOH, including inadequate education, limited employment opportunities, substandard housing, unsafe neighborhoods, poor transportation access, and environmental racism (113, 114, 117, 118). Financial insecurity not only constrains food access but also it creates a domino effect of vulnerabilities that compromise health, mobility, and autonomy (119).

Finally, the “double burden of malnutrition” (DBM), where undernutrition and overnutrition coexist within the same household or community, is increasingly evident among marginalized populations. While traditionally associated with low- and middle-income countries, DBM also appears in high-income nations (120, 121). For instance, a Brazilian study found that severe household food insecurity correlated with the presence of DBM, primarily because financial constraints forced families to rely on inexpensive, calorie-rich but nutrient-poor foods (122). Among older adults, this dual burden contributes to poor diet quality and heightened susceptibility to chronic diseases, reinforcing existing health disparities (121). While these findings underscore the importance of intersectional approaches, further research is needed to disentangle the complex interactions between social determinants, structural factors, and biological processes across diverse populations.

## 7 Mediators and moderators of nutritional aging

Mental health is a critical determinant of nutritional status in older adults. Those facing food insecurity are significantly more likely to report poor overall health and higher rates of mental health issues, particularly depression (123). Socioeconomic disadvantage, especially poverty and low SES, is associated with a higher risk of psychological disorders, as prolonged economic stress can trigger physiological changes that heighten vulnerability to depression (115). As life expectancy increases, the global burden of depression and dementia is expected to rise, given that aging itself is a primary risk factor for both conditions (124, 125). Epidemiological evidence suggests that malnutrition and depression frequently co- occur in older adults, with some studies reporting higher prevalence among men (126). A cross-sectional study conducted in Faridpur, Bangladesh, further supports this association this link, revealing high rates of malnutrition and depression among older adults, with a significant inverse relationship between nutritional status and mental well-being. These findings underscore the need for integrated interventions that address both nutritional status and mental health in older populations (127).

Beyond individual psychological factors, social isolation compounds these challenges. Around one in four community-dwelling older adults in the U.S. experiences social isolation, which is associated with poorer physical and mental health outcomes (128). Among seniors with low SES, loneliness has been identified as a key factor associated with functional deterioration, often affecting mobility and daily activities (128). Importantly, the relationship between mental health, socioeconomic status, and nutrition is bidirectional and self-reinforcing (129). Depression has been linked to food insecurity but may also worsen it by reducing motivation, impairing cognition, and limiting the ability to obtain or prepare nutritious food (130). This cycle of poor mental health and inadequate nutrition becomes particularly pronounced among socially isolated and economically disadvantaged older adults. Accordingly, comprehensive nutrition programs should integrate mental health care, include routine screening for depression and loneliness, and recognize food insecurity as a key driver of psychological distress (130, 131).

In addition to psychosocial determinants, physical and cognitive impairments are associated with older adults’ ability to access and consume nutritious food. Conditions such as dementia, vision loss, physical disabilities, and poverty make food acquisition and preparation more difficult (132, 133). Even when controlling for SES, physical limitations remain strongly associated with food insecurity (132). Moreover, declining health not only reduces mobility but also increases medical expenditures, further constraining food access (119). Physiological aging brings about reduced muscle mass, slower metabolism, diminished appetite and thirst, and increased vulnerability to digestive and dental issues. When combined with long-standing dietary habits and chronic psychological stress, these changes further increase the risk of malnutrition (132, 134, 135).

Among physical determinants, oral health is a particularly crucial but often overlooked aspect of nutrition in older adults. Conditions like periodontal disease is associated with chronic pain, infections, and tooth loss, all of which may impair food intake (136). Xerostomia, or chronic dry mouth further contributes to difficulty chewing, swallowing, taste impairment, and dental decay (136, 137). These complications not only limit dietary variety and quality but also interfere with denture use, exacerbating the risk of malnutrition (137). Addressing these challenges requires integrating oral and physical health assessments into nutrition interventions for older adults. Dental care, management of xerostomia, and supportive services such as home-delivered meals should be considered essential components of comprehensive geriatric care rather than optional services (136, 138).

In parallel, medication use adds another layer of complexity. Polypharmacy, commonly defined as taking five or more medications, is widespread among older adults, particularly in long-term care facilities (139). An umbrella review covering nearly 60 million individuals across 41 countries found that the global prevalence of polypharmacy is 37%, rising to 45% among older adults and up to 71% among hospitalized frail elderly, with regional variation (140). This high prevalence is associated with an increased risk of drug-nutrient interactions, particularly among individuals with existing nutritional deficiencies or chronic conditions (141).

Numerous medications contribute to nutrient depletion. For example, statins may reduce levels of coenzyme Q10 and fat-soluble vitamins (A, D, E, and K); proton pump inhibitors (PPIs) impair absorption of vitamin B12, calcium, magnesium, vitamin C, and iron; metformin depletes B12 and folic acid; and selective serotonin reuptake inhibitors (SSRIs) are linked to calcium and vitamin D loss (141, 142). These medication-induced deficiencies can exacerbate systemic health conditions, contributing to anemia, osteoporosis, cognitive decline, and oral health deterioration (139, 142).

## 8 Research gaps and future directions

A significant research gap remains in understanding how food insecurity is associated with long-term functional decline in older adults. While cross-sectional studies show associations, they are inherently limited in capturing the temporal dynamics and causal pathways underlying health disparities. Longitudinal studies, such as the recent 10-year SNAP study showing slower cognitive decline among recipients, highlight the value of tracking outcomes across the life course (143). Indeed, longitudinal evidence is critical for disentangling bidirectional relationships between food insecurity, financial stress, and health deterioration (144). Such data are essential for establishing causal relationships between food insecurity, financial stress, and health decline, especially among vulnerable groups. However, racial disparities in aging, particularly among Black populations, are under-researched due to the scarcity of long-term, diverse cohort datasets and the underrepresentation of minority groups in genomic and epidemiological research. Addressing these gaps requires the integration of longitudinal health records, biomarker data, and diverse population cohorts to uncover mechanisms underlying disparities in cognitive and functional aging.

In parallel, improving the measurement of socioeconomic vulnerability is essential. Tools like the Older Adult Socioeconomic Disadvantage Index (OASDI) provide a comprehensive approach. This multi-dimensional index captures unmet needs (food, housing, transport, healthcare) and incorporates factors such as race, education, poverty, disability, and social isolation (145). When applied at the neighborhood level, OASDI enables the identification of localized disparities and supports the development of targeted, place-based interventions. The broader adoption of standardized and actionable metrics such as OASDI could substantially improve resource allocation, policy planning, and program evaluation. Addressing nutritional disparities requires interdisciplinary approaches that integrate gerontology, public health, and nutrition science. The Nutrition Health Disparities Framework (NHDF) provides a useful model, analyzing biological, social, environmental, and systemic factors at multiple levels. Future research must move beyond documenting disparities to uncovering biological mechanisms, such as how racism accelerates aging, and to develop equity-oriented and mechanism-informed interventions. Finally, translating evidence into practice requires culturally responsive strategies. Traditional food practices among immigrant communities often promote healthier diets and support cultural identity. Public health interventions should move beyond standardized definitions of “healthy” foods and instead offer culturally appropriate and acceptable options. Engaging communities through participatory research ensures that interventions are relevant, respectful, and effective. Food extends beyond basic sustenance, encompassing cultural identity, social meaning, and lived experience, and should therefore be central to the design of nutrition interventions.

## 9 Conclusion

The evidence reviewed highlights that socioeconomic inequality is a key factor associated with nutritional decline and accelerated health deterioration. Food insecurity and financial hardship, compounded by structural determinants such as systemic racism, inadequate social safety nets, and inequitable access to affordable, nutritious food, create a self- reinforcing cycle that profoundly compromises health outcomes. These challenges are reflected in not only in rising rates of undernutrition and frailty, but also in cognitive decline and, paradoxically, increasing obesity among the most vulnerable seniors. While these associations are consistently reported, much of the evidence remains observational, and causal relationships should be interpreted with caution.

Achieving equitable and healthy aging requires a fundamental shift from descriptive to mechanism-oriented and intervention-driven approaches. Future efforts should move beyond documenting disparities to elucidating underlying biological and social mechanisms, while simultaneously developing structural, equity-oriented interventions. This includes the use of comprehensive tools like the Older Adult Socioeconomic Disadvantage Index (OASDI) to better capture the lived realities of older adults, and the implementation of culturally sensitive public health strategies that acknowledge food’s deep connection to identity, tradition, and community. Addressing the structural determinants of nutritional disparities is essential to ensuring that increased longevity is accompanied by improved healthspan, thereby reducing inequities in aging outcomes across populations. These disparities require integrated strategies that take into account the multidimensional nature of food security, including structural, environmental, and individual determinants.
